# Post-Traumatic Stress Disorder (PTSD) Resulting from Road Traffic Accidents (RTA): A Systematic Literature Review

**DOI:** 10.3390/ijerph22070985

**Published:** 2025-06-23

**Authors:** Marija Trajchevska, Christian Martyn Jones

**Affiliations:** Engage Research Lab, School of Law and Society, University of the Sunshine Coast, Sippy Downs, QLD 4556, Australia; marija.trajchevska@research.usc.edu.au

**Keywords:** post-traumatic stress disorder, PTSD, car accident, traffic accident, car crash, road accident

## Abstract

Road traffic accidents (RTAs) are a leading cause of physical injury worldwide, but they also frequently result in post-traumatic stress disorder (PTSD). This systematic review examines the prevalence, predictors, comorbidity, and treatment of PTSD among RTA survivors. Four electronic databases (PubMed, Scopus, EBSCO, and ProQuest) were searched following PRISMA 2020 guidelines. Articles were included if reporting on the presence of post-traumatic stress disorder as a result of a road traffic accident in adults aged 18 years and older. Including peer-reviewed journal articles and awarded doctoral theses across all publication years, and written in English, Macedonian, Serbian, Bosnian, Croatian, and Bulgarian, identified 259 articles, and using Literature Evaluation and Grading of Evidence (LEGEND) assessment of evidence 96 were included in the final review, involving 50,275 participants. Due to the heterogeneity of findings, quantitative data were synthesized thematically rather than through meta-analytic techniques. Findings are reported from Random Control Trial (RCT) and non-RCT studies. PTSD prevalence following RTAs ranged widely across studies, from 20% (using Diagnostic and Statistical Manual of Mental Disorders, Fifth Edition, DSM-5 criteria) to over 45% (using International Classification of Diseases, 10th Revision, ICD-10 criteria) within six weeks post-accident (non-RCT). One-year prevalence rates ranged from 17.9% to 29.8%, with persistence of PTSD symptoms found in more than half of those initially diagnosed up to three years post-RTA (non-RCTs). Mild or severe PTSD symptoms were reported by 40% of survivors one month after the event, and comorbid depression and anxiety were also frequently observed (non-RCTs). The review found that nearly half of RTA survivors experience PTSD within six weeks, with recovery occurring over 1 to 3 years (non-RCTs). Even minor traffic accidents lead to significant psychological impacts, with 25% of survivors avoiding vehicle use for up to four months (non-RCT). Evidence-supported treatments identified include Cognitive Behavioural Therapy (CBT) (RCTs and non-RCTs), Virtual Reality (VR) treatment (RCTs and non-RCTs), and Memory Flexibility training (Mem-Flex) (pilot RCT), all of which demonstrated statistically significant reductions in PTSD symptoms across validated scales. There is evidence for policy actions including mandatory and regular psychological screening post RTAs using improved assessment tools, sharing health data to better align early and ongoing treatment with additional funding and access, and support and interventions for the family for RTA comorbidities. The findings underscore the importance of prioritizing research on the psychological impacts of RTAs, particularly in regions with high incident rates, to understand better and address the global burden of post-accident trauma.

## 1. Introduction

Most individuals experience at least one potentially traumatic event (PTE) during their lifetime, which can lead to acute psychological symptoms such as severe anxiety, dissociative symptoms, and sleep disturbances. While not all individuals exposed to a PTE develop Post-Traumatic Stress Disorder (PTSD), symptoms can persist and worsen over time [1]. PTSD is characterized by re-experiencing, avoidance, negative cognitions and mood, and heightened arousal following exposure to a traumatic event [2]. PTSD is classified in the Diagnostic and Statistical Manual of Mental Disorders, Fifth Edition (DSM-5), as a trauma- and stressor-related disorder that may develop following exposure to actual or threatened death, serious injury, or sexual violence [1]. It is defined by symptoms across four domains: intrusive memories, avoidance, negative mood and cognition, and hyperarousal. While PTSD remains a formal psychiatric diagnosis, recent literature increasingly recognizes it as a complex, context-dependent response to trauma, particularly in cases of repeated or prolonged exposure such as road traffic accidents [3,4]. RTAs are among the most common global trauma exposures and represent a significant cause of PTSD, particularly in countries with high rates of traffic-related injuries. PTSD prevalence following RTAs has been reported between 20% and 45% in the initial weeks after the incident, with symptoms persisting in a substantial proportion of cases up to three years later [5,6,7]. Despite this, the psychological burden of RTAs remains underrepresented in the trauma literature, especially in low- and middle-income countries [2,8].

In recent years, a growing number of studies have examined the psychological consequences of RTAs, with a particular emphasis on the development and trajectory of PTSD symptoms. Previous systematic reviews have addressed specific aspects of this issue, however, often within a narrow focus. For instance, Lin et al. [9] reported on prevalence rates but did not explore factors associated with symptom severity or long-term recovery. Ponniah and Hollon [10] provided a broader review of PTSD treatments, but without consideration of RTA-specific populations. Boccia et al. [11] focused on hospital-based Eye Movement Desensitization and Reprocessing (EMDR) treatment, calling for further research involving outpatient populations or those with less severe physical injuries.

Previous systematic reviews have addressed various aspects of PTSD following RTAs, but gaps remain. For example, Lin et al. [9] focused on prevalence but not on PTSD severity or predictors, while Ponniah and Hollon [10] emphasized treatment without considering the post-RTA quality of life. Boccia et al. [11] concentrated on hospital treatments, recommending further research on PTSD in individuals with minor injuries.

Furthermore, studies from low- and middle-income countries are still underrepresented in global syntheses, despite high rates of RTAs in these regions [12,13]. Moreover, many existing reviews exclude research published in languages other than English, limiting the diversity of perspectives and experiences considered in the field. The review included studies conducted in underrepresented countries, including those in Southeastern Europe, to address regional gaps, even if published in English. This allowed for greater global representation and inclusion of data from regions often excluded in prior reviews. Road traffic accident (RTA) fatality rates in Southeastern Europe—specifically in North Macedonia, Serbia, Bosnia and Herzegovina, Croatia, and Bulgaria—remain above the European Union average, reflecting persistent road safety challenges in these countries [14]. To strengthen its contribution to the literature, the review includes studies from underrepresented low- and middle-income countries, incorporates populations with minor injuries, and synthesizes findings across a wide range of validated PTSD assessment tools. This systematic review aims to explore PTSD among adult road traffic accident survivors through a narrative thematic synthesis, with key outcomes, including prevalence, severity, predictors, comorbidity, and treatment, emerging from the literature rather than being defined a priori. The review aims to provide a practical understanding of service delivery, particularly in lower-resource settings often underrepresented, and promote actionable and achievable changes to improve the lives of those surviving a RTA.

## 2. Materials and Methods

This systematic review was conducted to provide an overview of the extant literature regarding people who developed symptoms of PTSD after surviving an RTA. The research followed the PRIMSA framework [15].

### 2.1. Information Sources

On 20 May 2024, the following databases were systematically searched: PubMed, Scopus, EBSCO, and ProQuest. The employed search strategy used the argument: (“Post-traumatic stress disorder” OR “PTSD”) AND (“car accident” OR “traffic accident” OR “car crash” OR “road accident”) in paper titles and abstracts. In addition, the references of review articles were examined for relevant publications.

### 2.2. Eligibility Criteria

Articles were included if they reported on the presence of post-traumatic stress disorder as a result of a road traffic accident in adults aged 18 years and older. Articles were considered should they meet search terms of: (“Post-traumatic stress disorder” OR “PTSD”) AND (“car accident” OR “traffic accident” OR “car crash” OR “road accident”). A total of 259 records were identified. After removing 67 duplicate records, 192 records remained for screening. The study included peer-reviewed journal articles and awarded doctoral theses across all publication years written in English, Macedonian, Serbian, Bosnian, Croatian, and Bulgarian to increase inclusivity and capture research from underrepresented regions. This multilingual approach enables a broader examination of global PTSD experiences following RTAs. The grey literature and unpublished studies were excluded to ensure the review included only peer-reviewed sources, maintaining consistent methodological quality. While the grey literature can be valuable in public health, this focus was on reliable, vetted evidence.

### 2.3. Data Screening and Extraction

**Figure 1:**
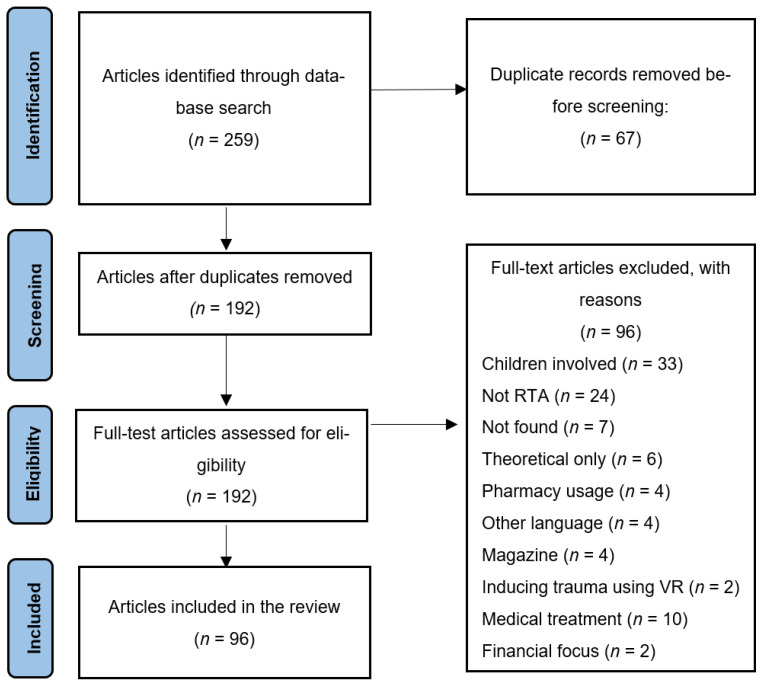
Using the PRISMA framework, PubMed, Scopus, EBSCO, and ProQuest were searched on 20 May 2024 using the search terms (“Post-traumatic stress disorder” OR “PTSD”) AND (“car accident” OR “traffic accident” OR “car crash” OR “road accident”). A total of 259 records were identified. After removing 67 duplicate records, 192 records remained for screening. During title and abstract screening, 96records were excluded for reasons including children involved (*n* = 33), not road traffic accident-related (*n* = 24), articles not found (*n* = 7), theoretical papers only (*n* = 6), pharmacy usage (*n* = 4), other language publications (*n* = 4), magazine articles (*n* = 4), studies inducing trauma via VR (*n* = 2), medical treatment focus (*n* = 10), and financial focus (*n* = 2). After full-text assessment, 96 articles were included in the final systematic review.

The identification stage involved retrieving articles from the search strategy, uploading them into EndNote and removing duplicates, Figure 1. In the screening phase, the two authors worked as independent reviewers assessing the 259 studies based on their titles and abstracts, with 67 duplicates removed, 96 articles removed due to their lack of relevance, such as articles about pharmacy usage (*n* = 4), theoretical articles (*n* = 6), other languages (*n* = 4), children involved (*n* = 33), articles published in magazines (*n* = 4), articles about medical treatment (*n* = 10), not RTA (*n* = 24), articles about inducing trauma using VR (*n* = 2), articles focused on financial impacts (*n* = 2), with seven articles not found.

Each reviewer independently assessed all articles across the title, abstract, and full-text phases. Discrepancies were resolved through consensus discussion between the reviewers. This independent review followed by consensus is consistent with the Preferred Reporting Items for Systematic Reviews and Meta-Analyses (PRISMA 2020) guidelines [16] and was used to ensure data extraction accuracy and reduce bias.

Of interest were studies regarding post-traumatic stress disorder as a consequence of road traffic accidents, excluding articles reporting post-traumatic stress disorder following other traumatic events. The severity of the injuries of the participants after the road traffic accidents was a focus and not the medical procedures or pharmacy products used in the treatment of injuries.

The process yielded 96 unique publications for analysis, including four doctoral theses, and diverse regions across continents, with authorship spanning Europe, Asia, Africa, North America, and Oceania. Empirical support for including doctoral theses/dissertations in a search lies in their original research, comprehensive coverage, and rigorous review processes. After screening, data were extracted independently by one reviewer and reviewed by the second reviewer. Discrepancies were resolved through discussion to ensure accuracy and consistency of the extracted data.

A standardized data charting sheet, including the Literature Evaluation and Grading of Evidence (LEGEND) assessment of evidence table [17], was created to extract relevant information from the selected. The charting sheet included the article title, publication year, authors, research methodology and study design, study area, demographic information and number of participants, study objectives, findings, conclusions, and limitations. PTSD-specific variables extracted included diagnostic criteria (e.g., DSM-IV or DSM-5, ICD-10, as used in the articles), assessment tools used, symptom severity, comorbidity, and treatment-related outcomes.

The LEGEND tool, developed for evaluating evidence in health outcomes research, was also used to assess methodological quality and potential risk of bias. All included studies met or exceeded a ‘moderate’ grading for LEGEND, in accordance with PRISMA 2020 guidelines, Table 1. The LEGEND quality ratings were used to support the interpretation of results by giving greater consideration to higher-quality studies during the thematic synthesis.

### 2.4. Data Synthesis

Articles were evaluated using the LEGEND assessment of evidence [104], followed by a thematic qualitative analysis [17]. A narrative synthesis was conducted using thematic analysis. This approach was chosen due to the heterogeneity of study designs, populations, outcomes measured, and reporting styles, which made meta-analysis inappropriate. The search strategy was open-ended and included studies from all publication years, without date restrictions. The characteristics of all studies were summarized in tabular form and presented in coherent theme areas in the results.

Thematic analysis allowed conceptually distinct but interconnected domains of predictors, measurement tools, and treatment modalities to be considered, generating understandings that can guide future research and practice [105]. Where evidence is still evolving, such as the use of Virtual Reality for PTSD or the psychological consequences of minor RTAs, a qualitative synthesis supports exploratory insights and identifies gaps in the literature. PRISMA 2020 guidelines endorse using qualitative systematic reviews and thematic synthesis as valid approaches where quantitative aggregation is not feasible or appropriate.

## 3. Results and Discussion

This systematic review provides a broader and more inclusive synthesis than previous reviews by incorporating studies from diverse geographic regions and languages, examining outcomes in both major and minor injury populations, and using a wide range of validated PTSD assessment tools. This approach enabled the exploration of predictors, comorbidities, and treatment responses not fully addressed in earlier systematic reviews.

Ninety-eight articles were purposefully examined using the LEGEND evidence evaluation for study methodology, participants, findings, and limitations, and all 96 articles were retained for inclusion in the review. Statistical significance, coupled with experimental limitations, are reported as measures of the quality of the extent of research and findings.

### Study and Sample Characteristics

Unlike previous systematic reviews that focused primarily on English-speaking, high-income countries, the current review incorporated studies across multiple languages and global regions, offering a more internationally representative synthesis of PTSD following RTAs. The first authors of the included articles were from 24 countries, primarily the United States (22 studies; 22.9%) and the United Kingdom (13 studies; 13.5%), Table 1. The first author’s country was used as a proxy for the geographical distribution and temporal trends of research on post- Road Traffic Accident (RTA) psychological effects. The focus has primarily been within the United Kingdom, the United States, and Europe. There is a notable absence of research in countries with high numbers of vehicles and annual traffic fatalities, such as Brazil (96 million road users, 33,800 road fatalities), Russia (58 million road users, 17,500 road fatalities), and Indonesia (22.5 million road users, 33,700 road fatalities) [106].

Table 1 outlines the characteristics of the included studies, presenting study design, primary methodology, and country of origin in alphabetical order. Table 2 summarizes the geographic and temporal distribution of studies, showing that the United States of America contributed the highest number of studies (22.9%) and that over half of the research (55.1%, *n* = 54) was published between 2011 and 2024. The earliest study dates back to 1989, reflecting a growing recent focus on PTSD following road traffic accidents.

The studies included 50,275 participants, Table 3. The greater proportion of studies, *n* = 77 (80.2%), did not report whether the participants were informed of, recommended to avail of, or had participated in psychological treatments after the Road Traffic Accidents and only *n* = 21 (21.8%) of the articles included treatments after an accident and reported the effects of the treatments. More studies involved participants not hospitalized after the RTA (67 studies, 69.8%) than those with participants hospitalized due to injuries from the accident (31 studies, 32.3%), Table 3. There is a significant gap in the literature, with the majority of studies not reporting whether participants received any form of psychological treatment following the accident. Additionally, more studies focused on non-hospitalized individuals than hospitalized participants, indicating potential disparities in research attention towards different severity levels of RTA injuries.

The most utilized scales in the articles were the PTSD-Check list PCL-5 (27 studies, 28.1%), the Clinical Administrated PTSD Scale (26 studies, 27.1%), the Impact of Event Scale (16 studies, 16.7%), the PSS-I scale (17 studies, 17.7%), and the Beck depression and anxiety inventories (9 studies, 9.4%), Table 4 and Table 5.

## 4. Overview of Findings

The reviewed literature considers people who experienced symptoms of post-traumatic stress disorder (PTSD) caused by a road traffic accident (RTA). The discussion frames around the prevalence of symptoms of PTSD, predictors of PTSD, management of thoughts, quality of life, post-traumatic growth, comorbidity of PTSD, treatments for PTSD, and the scales used to measure PTSD.

### 4.1. PTSD Symptom Prevalence and Persistence

#### 4.1.1. Prevalence of Symptoms of PTSD

After a car crash, nearly half of RTA survivors develop PTSD. Between 1 in 5 (using Diagnostic and Statistical Manual) and nearly half (using International Classification of Diseases, 10th Revision, ICD-10) of people experience PTSD (DSM-5, 20% and ICD-10, 46%) at six weeks post-RTA (*n* = 69, 18–65 years) [5]. PTSD in the first year following the RTA was reported at 45.2% (*n* = 274) [6]. Similarly, one month after their RTA, 40% of 5807 people had PTSD at mild (28.6%) or severe (11.6%) levels [95]. Road survivors (*n* = 155, median age = 42 years, and 54.8% male) one month after their RTA reported PTSD (32.3%), depression (17.4%), and anxiety (5.8%) [75]. Similar studies report PTSD prevalence at 41% (*n* = 46) for survivors of RTAs compared with 13% (*n* = 46) for controls, and depression among RTA survivors at 63% compared with 34% among the controls [21,38]. Eighteen per cent (*n* = 592) of people six months post-accident had PTSD [45], and PTSD affected 29.8%, 23.1%, and 17.9% (*n* = 95) of people 3, 6, and 12 months after a road accident, respectively [102]. Furthermore, even minor RTAs can lead to clinically significant psychological distress [20,142], and time since the accident, having depressive symptoms, history of a previous RTA, and common mental disorders are significant determinants of PTSD [29].

#### 4.1.2. Persistence of Symptoms of PTSD

Within a year, the prevalence of PTSD was 22.6% (*n* = 300) [92]. After one year, 7% still had post-traumatic stress disorder and 9% had subsyndromal post-traumatic stress [67]. Over half of those who had PTSD still had it three years post-accident [8,63]. Interestingly, seven years after the accident, eight people (1.0%) out of 774 participants met the criteria for PTSD [63]. However, remission is possible and was observed after a one-year follow-up for 77% (*n* = 48) of people who initially met the criteria for PTSD [32].

### 4.2. Risk Factors for Developing PTSD Post-RTA

#### 4.2.1. Biological and Injury-Related Factors

The severity of injuries immediately after an RTA significantly positively correlates with PTSD severity at four weeks (*r* = 25, *p* < 0.01) and at six months (*r =* 0.21, *p < 0*.05), based on a longitudinal study using patients’ self-rated injury severity [85]. The type and severity of injury was identified as the leading risk factor for the development of PTSD (*p =* 0.013), with a PTSD prevalence of 54.2% among 120 participants in a cross-sectional study [83]. In a longitudinal study, individuals involved in an RTA (*n =* 1260) who had been unconscious (22.8%) or had major injuries (9.5%) more commonly developed PTSD, depression, and anxiety than those without such injuries [93]. However, another longitudinal study found that one year post-accident, there were no significant differences in PTSD development between individuals who had been unconscious and those who had not [82]. In contrast, a cross-sectional study found no association between physical injury severity and PTSD symptom severity [24]. Additionally, post-RTA pain (*p =* 0.006) and duration of hospitalisation (*p =* 0.011) were also significantly associated with PTSD in a cross-sectional study [75].

#### 4.2.2. Psychological Factors

Several cross-sectional studies have identified psychological risk factors for PTSD. These include self-perceived threat to life (*p =* 0.002), previous psychiatric illness (*p =* 0.008), and psychiatric medication use (*p <* 0.001) [76]. PTSD symptom severity was also significantly higher in females than males (*p <* 0.05), and in married individuals compared to singles (*p <* 0.001) [79]. Additionally, a significant psychological finding was that forgiveness of the person at fault for the accident reduced PTSD severity (*β* = −0.21, *t* = −3.50, *p <* 0.001) [64]. Although survivors with minor injuries may not appear to be at high risk, research suggests they are not immune to PTSD; however, little is known about the long-term severity or impact of their symptoms.

#### 4.2.3. Social and Demographic Factors

A cross-sectional study found that key sociodemographic factors significantly associated with PTSD included female gender (*p =* 0.038), below-average self-perceived economic status (*p =* 0.025), and general medication use (*p =* 0.002) [75]. Other social factors included being not at fault in the RTA (*p =* 0.044) and having lodged a compensation claim (*p <* 0.001). In a longitudinal study, 20% of severely injured participants reported relational difficulties with their partner, 16% reported impaired sexual life, and separation rates were significantly higher among those severely injured compared to those with mild-to-moderate injuries [64].

### 4.3. Sociodemographic Factors Do Not Impact PTSD Post-RDA

There is some inconsistency in findings on gender and PTSD. Some have found gender to be a predictor for increased prevalence [12,71] and severity of PTSD developed after an RTA [56,75,83,88,107]. However, others have not found a significant relationship between the severity of PTSD and the gender of RTA survivors [9,21]. Similarly, there was no significant relationship found between the development of PTSD and the country of origin, race [78], and education level [71,78] of RTA survivors. There was no significant correlation between the severity of PTSD and the age of the people who had road collisions [21,59]. Sociodemographic characteristics such as marital status, religion, level of education, and professional occupation of people who had RTA did not show a significant relationship with PTSD or depression [21].

### 4.4. Cognitive Mechanisms Associated with PTSD After an RTA

Difficulties in managing trauma-related thoughts and cognitive processes following a road traffic accident (RTA) are associated with the development and persistence of PTSD. Key mechanisms that may influence symptom expression include rumination, thought suppression, working memory deficits, maladaptive beliefs about thoughts, and impaired coping strategies. While these factors have not all been evaluated in clinical trials, they represent cognitive correlates and modifiable risk processes that are relevant to understanding post-RTA PTSD.

#### 4.4.1. Rumination and Thought Suppression

Rumination has been consistently identified as a factor that maintains trauma-related emotional disorders. Repetitive and involuntary focus on trauma-related thoughts has been observed in individuals with PTSD following an RTA [28,51,80,93,122]. Deliberate suppression of such thoughts may also be counterproductive, e.g., suppression of thoughts about the RTA has been found to result in increases in trauma-related intrusions, even in individuals who did not meet the criteria for PTSD [27]. These findings indicate that both rumination and suppression are cognitive responses that may exacerbate trauma symptoms.

#### 4.4.2. Working Memory and Cognitive Interference

Impairments in working memory and cognitive control appear to influence the severity of PTSD symptoms. An indirect relationship has been identified between PTSD diagnosis and working memory capacity in emotionally charged contexts, mediated by re-experiencing symptoms and avoidance behaviors such as evading distressing thoughts and emotions [49]. Individuals with reduced working memory capacity may be less able to manage trauma-related cognitive demands. Further evidence shows that individuals with PTSD experience significant impairments in proactive interference, whereby older memories interfere with the recall of newer ones [120]. These types of cognitive disruptions may impair the integration of new, non-threatening information following trauma.

#### 4.4.3. Metacognitive Beliefs

Beliefs about one’s thoughts and the ability to control them have also been linked to PTSD severity. PTSD symptoms have been found to correlate significantly and positively with positive beliefs about worry (*r* = 0.25, *p* = 0.006), beliefs about controlling thoughts (*r* = 0.50, *p* < 0.0005), and negative beliefs regarding the perceived danger of certain thoughts (*r* = 0.60, *p* < 0.01) [94]. These findings highlight the role of maladaptive metacognitive beliefs in the maintenance of PTSD symptoms, particularly when individuals interpret intrusive thoughts as dangerous or uncontrollable.

#### 4.4.4. Symptom Pattern and Timing

Re-experiencing and avoidance symptoms do not always emerge with equal prominence in the early stages of PTSD. At six weeks post-RTA, 74 ± 4% of participants were found to meet the criteria for re-experiencing symptoms, whereas only 38 ± 5% met the criterion for avoidance and emotional numbing (*n* = 45) [86]. These findings suggest that re-experiencing symptoms are more likely to be reported in the acute phase following trauma, while avoidance-related responses may be less immediately evident or more difficult to detect.

#### 4.4.5. Coping Style and Interpersonal Functioning

The capacity to cope with everyday stressors may also be compromised in individuals who have experienced trauma. Individuals exposed to traumatic events, including RTAs and physical assault, have been shown to report reductions in both individual and dyadic coping when compared to non-traumatized controls [76]. These impairments in coping may limit both internal emotional regulation and interpersonal functioning, potentially contributing to the persistence of PTSD symptoms [44]. Although these findings do not derive from intervention trials, they point to the broader psychosocial impacts of trauma on cognitive and relational processes.

### 4.5. Quality of Life and PTSD

The presence of PTSD can reduce quality of life. Low scores in all (physical health, psychological health, social relationships, and environmental health) WHOQOL-BREF domains (The World Health Organization Quality of Life Questionnaire-Brief Version) [125] were linked with PTSD (physical health *B* = −2.05; *SD* = 0.28; *p* < 0.0001, psychological health *B* = −2.28; *SD* = 0.31; *p* < 0.0001, social relationships *B* = −1.76; *SD* = 0.42; *p* < 0.0001, environmental health *B* = −1.83; *SD* = 0.30; *p* < 0.001) diagnosed in participants who had road traffic accidents, as well as socioeconomic characteristics (age/physical Health *B* = −0.03; *SD* = 0.21; *p* < 0.01) and financial problems (physical health *B* = −1.05; *SD* = 0.25; *p* < 0.0001, environmental health *B* = −1.26; *SD* = 0.25; *p* < 0.0001) [72]. Individuals with PTSD performed significantly poorer memory flexibility than both trauma-exposed control participants (*p* = 0.006, d = 0.69, 95% CI = [0.04, 1.34]) and community control participants (*p* < 0.001, d = 1.59, 95% CI = [0.87, 2.31]) [89]. The RTA had a negative impact on the survivor’s monthly income (*n* = 555, PTSD M = 181, 32.6%) [11], PTSD sufferers after an RTA reported significantly lower scores in time management (PTSD *M* = 6.92, *SD* = 2.19, non-PTSD *M* = 9.28, *SD* = 1.36; *F*(1.39) = 17.59, *p* = 0.000), and preoccupation with health (PTSD *M* = 5.75, *SD* = 2.14, non-PTSD *M* = 8.45, *SD* = 1.74; *F*(1.39) = 17.80, *p* = 0.000) [81], and experienced extreme impact on work or school (70.2% of PTSD sufferers) and (51.9%) problems in social life [55]. Research involving university students who read vignettes describing persons with PTSD who varied by the trauma they experienced (*n* = 560, *n* = 407 women, *n* = 152 men, age range = 18–25 years) revealed that rape as a precipitating traumatic event resulted in a lower perceived stigma of the person than compared to a car accident [77].

### 4.6. Post-Traumatic Growth After a RTA

No significant relationship was found between PTSD and Post-Traumatic Growth (PTG) [22]. However, situations exist where the presence of PTSD can result in growth in psychological self-perception, well-being, and greater optimism. Greater post-traumatic growth was more likely in married and more educated RTA survivors [112]. PTG was not significantly linked to age, gender, and enduring time since the RTA [124], however associations were found between PTG sub-dimensions and PTSD [112], including significant negative correlations with personal strength (r = −0.20, *p* < 0.05) and significant positive correlations with the appreciation of life (r = 0.28, *p* < 0.01) and spiritual change (r = 0.20, *p* < 0.05) [103]. Significantly higher scores were reported by PTSD-diagnosed persons involved in RTA on the extrinsic motivation scale (PTSD *M* = 4.17, *SD* = 1.53, non-PTSD *M* = 2.42, *SD* = 1.21) [81]. For RTA survivors with high PTSD severity, greater optimism was related to higher reports of growth [103]. However, individuals in highly traumatic RTAs with major injuries may have received more social support than persons with minor injuries. Thus, any personal growth may have been fostered by increased social support [103].

### 4.7. Comorbid Psychological and Cognitive Factors Associated with PTSD Post-RTA

Research indicates that post-traumatic stress disorder (PTSD) following a road traffic accident (RTA) often co-occurs with other psychological and cognitive conditions, including depression, acute stress disorder (ASD), anxiety, and the effects of traumatic brain injury (TBI). These comorbidities may influence both the onset and severity of PTSD symptoms and can complicate assessment and treatment.

#### 4.7.1. Acute Stress and Mood Disorders

PTSD after an RTA is significantly associated with current depression (*χ*^2^ = 53.25, *p* < 0.001) and with length of amnesia (*KW* = 6.23, *p* = 0.01) [73]. ASD has been shown to predict PTSD severity, with nearly one-fifth of RTA survivors experiencing ASD symptoms, including mood disturbance and intrusive memories [68,78,102]. One study found that 41.1% of participants had ASD, with higher risk associated with lower perceived social support (Odds Ratio = 0.0908, 95% CI = 0.834–0.989, *p* = 0.027) and higher peri-traumatic dissociation (*Odds Ratio* = 1.332, 95% *CI* = 1.170–1.516, *p* < 0.001) [102]. Although symptoms of anxiety and depression typically improved over time, approximately 10% of individuals continued to experience mood disorders one year after the RTA [84,143].

Peri-traumatic distress has also been identified as a relevant predictor of PTSD. The Peri-traumatic Distress Inventory (PDI) was strongly correlated with scores on the Impact of Event Scale—Revised (IES-R) (*p* < 0.05) [19], as well as with the PTSD Checklist (*p* = 0.008) [108] and the Clinician-Administered PTSD Scale (CAPS) (*p* = 0.03) [144]. These findings suggest that peri-traumatic emotional responses play a critical role in determining PTSD outcomes.

#### 4.7.2. Traumatic Brain Injury and PTSD Symptom Profiles

The presence of traumatic brain injury (TBI) can influence the manifestation of ASD and PTSD symptoms. In individuals assessed six days after an RTA, those with TBI showed less recall of fear at the time of the accident, more dissociative symptoms since the event, and fewer recurrent intrusive thoughts and images compared to those without TBI [68]. At six weeks post-accident, individuals with TBI also exhibited less re-experiencing and fear-related symptoms than those without TBI. Additional findings indicate that ASD, chronic PTSD, and TBI are associated with distinct patterns of disorganised narrative memory regarding the accident [43,69].

#### 4.7.3. Comorbidity with Other Psychological and Cognitive Factors

PTSD has been found to co-occur with a range of psychological and cognitive issues. Comorbidity was significantly associated with PTSD (Adjusted Odds Ratio = 2.29, 95% CI: 1.28–4, *p* < 0.05) [13,18]. Involvement in RTAs with fatal outcomes has been linked to high levels of self-blame, including self-reproach for the cause of death (*r* = 0.42, *p* < 0.01), belief that the accident could have been prevented (*r* = 0.28, *p* < 0.05), and generalized blaming of others (*r* = −0.47, *p* < 0.05) [79]. Anxiety has also been reported following minor RTAs [57], and high hyperarousal scores on intrusion and avoidance measures have been associated with increased risk of PTSD [65].

Cognitive functioning in RTA survivors with PTSD may also be impaired by co-occurring depressive symptoms. One study found that depressive symptoms negatively impacted immediate verbal recall (*r* = −0.56, *p* < 0.001), delayed recall (*r* = −0.38, *p* < 0.05), and delayed recognition (*r* = −0.55, *p* < 0.001), although these findings were based on a small sample (*n* = 24) and may have been influenced by medication use during assessment [23]. Additionally, PTSD was associated with increased self-reported sadness and more frequent facial expressions of sadness in affected individuals [42].

Among women who had experienced RTAs, PTSD was found to be related to self-esteem, resilience, and negative post-traumatic cognitions related to self-evaluation, memory, attention, planning, and problem-solving [70]. These associations persisted even after accounting for the overall severity of PTSD symptoms suggesting that post-traumatic self-perceptions may represent an important domain of comorbidity that warrants further investigation.

### 4.8. Treatments for PTSD

#### 4.8.1. Cognitive Behavioural Therapy for PTSD Post-RTA

Cognitive Behavioural Therapy (CBT) variants have been used to reduce post-traumatic stress disorder and have a positive impact on the mental health of the survivors of road traffic accidents ([53,66,96], RCT; [54], non-RCT; [10], review of RCTs). CBT (*n* = 30) had significant reductions in Clinician-Administered PTSD Scale (CAPS) [109] scores compared to a support group (*n* = 27) and waitlist (*n* = 22) for RTA survivors ([33], non-RCT). Patients completing psychotherapy (12-week session of Cognitive Behavioural Therapy) had reductions in Anxiety Sensitivity (r = 3.02, *p* < 0.01) and Pain Severity (r = 3.17, *p* < 0.01) ([54], non-RCT). At two-year follow-up for people who had survived an RTA and developed PTSD (*n* = 100), no significant differences in PTSD severity (Clinician-Administered PTSD Scale) were found between treatments involving brief exposure (CBT-B) to trauma memories (10 min exposure) or prolonged exposure to trauma memories (40 min exposure) (CBT-L) after a twelve-week therapy period [41]. Significantly more RTA survivors with PTSD experienced improvements after a four-week Brief-CBT intervention than those in a self-help booklet program (SHP) as measured using the Hospital Anxiety and Depression Scale (HADS)-D and the Impact of Events Scale (IES)- Hyperarousal scale from one-month pre-treatment to six-month post-treatment ([113], RCT). Regarding psychotherapy treatments, three in four people who suffered in Road Traffic Accidents (*n* = 269) preferred personal (face-to-face) care, and less than one in 10 preferred some other form (e.g., via the Internet) ([36], non-RCT).

#### 4.8.2. Virtual Reality Treatment for PTSD Post-RTA

Virtual Reality Treatment positively impacted the psychological condition of people who were exposed to a traumatic event ([25], RCT; [27,99], non-RCT), with reductions in reported post-trauma symptoms of re-experiencing (*p* < 0.05), avoidance (*p* < 0.05), and emotional numbing (*p* < 0.05) ([27], non-RCT). Furthermore, participants following an RTA and meeting DSM-IV criteria for Simple Phobia/Accident Phobia were exposed to a Virtual Driving Environment (Hanyang University Driving Phobia Environment) and computer driving games (London Racer/Midtown Madness/Rally Championship). Participants who experienced anxiety in one of the driving simulations took part in a cognitive behavioural program (12 sessions × 1 h, including driving simulation tasks), which resulted in significant post-treatment reductions in all measures: Subject Units of Distress Scale [30] (*p* = 0.008), Fear of Driving Inventory (FDI) ([99], non-RCT) (*p* = 0.008), Clinical Administrated PTSD Scale [145] (*p* = 0.008), Heart Rate (*p* = 0.008), and Hamilton Depression Scale [110] (*p* = 0.031). Further analysis of the FDI showed significant reductions in all three subscales: travel distress (*p* = 0.008), travel avoidance (*p* = 0.008), and maladaptive driving strategies (*p* = 0.016) ([99], non-RCT), suggesting that VR and driving games may reduce driving phobia post-accident even when co-morbid conditions such as post-traumatic stress disorder and depression are present. Using Virtual Reality Exposure Therapy (VRET) with twenty-six patients with PTSD (19 motor vehicle accidents and eighteen subjects without PTSD) and controlling for age, gender, marital status, job, economic status, and body mass index, found that the Clinical Global Impression-Severity scale (CGI-S) (*F* = 12.76, *p* = 0.001), Post-traumatic Stress Disorder Checklist-Civilian version (PCL-C) (*F* = 11.87, *p* = 0.002), Impact of Event Scale-Revised (IES-R) (total score; *F* = 8.31, *p* = 0.007), and Sheehan Disability Scale (SDS-A) (*F* = 7.53, *p* = 0.010) scores in the VRET group were lower than those in the control group ([74], non-RCT).

#### 4.8.3. Other Treatments of PTSD Post-RTA

At follow-up, a version of Mem-Flex called the Memory Structured Intervention (MSI) helped patients who survived an RTA to significantly experience less frequent total PTSD *t*(13) = 2.36, *p* < 0.05, less frequent intrusion symptoms *t*(10) = 2.50, *p* < 0.05 and less frequent arousal symptoms *t*(15) = 1.90, *p* < 0.05 ([60], pilot RCT) post-intervention compared to pre-intervention. Positive emotion while performing a dual task may be another practical therapeutic approach. Participants exposed to a clip with a positive context reported that their trauma memory was less distressing (not less vivid) than those exposed to a neutral context ([120], non-RCT). On the other hand, writing about a stressful event as a treatment for surviving trauma soon after the accident may lead to more anxiety (compared to completion of a non-recall task) ([61], RCT). Participants who suffered in an RTA and had been diagnosed with PTSD appreciated counselling as a treatment, but unfortunately, the counselling was not effective in preventing disorders [39]. For example, cognitive reconstruction of the event by vividly imagining the feared situation, known as ‘imaginal exposure’ (IE), significantly reduced PTSD (*p* < 0.05) compared with supportive counselling at post-treatment and 6-month follow-up ([41], RCT). Also, the arts program consisted of writing and drawing with PTSD severity assessed at 2, 6, and 12 months post-injury using clinical interviews (Clinician-Administered PTSD Scale, CAPS), self-report instrument (Impact of Event Scale-Revised, IES-R), and secondary outcomes were post-traumatic growth (PTG), depression and anxiety symptoms. Repeated measures analysis of variance indicated that the Arts program and the control group exhibited a significant effect of time (*p* < 0.01) on PTSD but no significant group differences ([100], RCT).

### 4.9. The Consequence of the PTSD

#### 4.9.1. Impact of PTSD Post-RTA on Heart Rate

Significant differences in the heart rate (HR) among the participants while resting and during a stressful event (Heart Rate Reactivity) were discovered between groups diagnosed with chronic PTSD and those with subsyndromal PTSD and non-PTSD [31,35,90,91,98]. Results showed a significantly greater reduction in HR reactivity for those receiving CBT (r = −0.217; *p* < 0.05) than those receiving Supportive Psychotherapy (*p* < 0.05; r = 0.487) [33]. There was no correlation between HR reactivity or gender with PTSD post-RTA [37].

#### 4.9.2. Impact of PTSD Post-RTA on Brain Function

Psychophysiological responses are generally diminished at a one-year follow-up for those with diagnosed PTSD compared to 1 to 4 months after the RTA [34]. Compared to the trauma victims without PTSD, those with PTSD had decreased fractional anisotropy values (quantification of brain white matter integrity) in the corpus callosum (large bundle of more than 200 million nerve fibres responsible for the communication between the right and left sides of the brain) (victims with PTSD = 0.58 ± 0.04; victims without PTSD = 0.61 ± 0.03; *t* = 2.137, *p* = 0.042), and no significant differences were detected for fibre length (victims with PTSD = 60.15 ± 4.66; victims without PTSD = 61.83 ± 4.6; *t* = 0.974, *p* = 0.339) [97]. Higher functional connectivity was found between the amygdala and somatosensory areas during trauma-specific stimulus presentation for an RTA group as compared to neutral stimuli [87] at 3 weeks post-traffic accident, and a cluster of activation was found in the right anterior cingulate cortex in people with PTSD [11].

#### 4.9.3. Impact of PTSD Post-RTA on Employment

RTA and the presence of PTSD can also affect employment. Multivariate analyzes showed that injury severity (23.7% for work-related accidents, *p* = 0.007) is important in determining the time to return to work [58]. Thirty-two per cent of the severe injury group (*p* < 0.001) who had stopped work following their RTA had not returned after one year post-accident [64]. PTSD is also associated with impairments in completing the job [26], and limitations at work and social life were predictors of PTSD 12 months after the accident [102]. A study reported poor quality of life and health status three years post-RTA as factors associated with non-return to work [90]. However, gender is not a factor, and no relationship was found between gender and changes in employment status after an RTA [48].

### 4.10. Scales Used to Measure PTSD

The Chinese version of the PTSD Checklist has been confirmed to have satisfactory reliability and validity [50,102], and The Depression Anxiety Stress Scales (DASS-21) and the Impact of Event Scale-Revised (IES-R) have acceptable sensitivity and specificity for detecting Major Depressive Disorder and PTSD in an injured population engaged in compensation [62]. The PTSD PK Scale [114] and PTSD PS Scale [52] are considered indices of general emotional distress and maladjustment. However, both scales failed in productive functional classification in diagnosing PTSD [49]. The Glover Numbing Scale (GNS) (a 35-item questionnaire intended to measure subjective experiences associated with Post-traumatic Numbing, one of the symptoms of PTSD) [46,146] was a poor fit for a sample of RTA survivors [47]. However, measures are imperfect as 25% of participants have been able to fake both the direct and indirect symptoms of psychological injury from a road traffic accident in their psychological evaluation [147].

## 5. Implications for Mental Health Services and Public Health Policy

There is an urgent need for enhanced mental health services and trauma care protocols for road traffic accident (RTA) survivors. With nearly half of the RTA survivors experiencing post-traumatic stress disorder (PTSD) following an accident [5,6,7,63,67,92], mental health services must integrate routine psychological screening as a standard part of post-accident care. Implementing evidence-based screening tools [101,127] in emergency departments and trauma units could facilitate early identification of PTSD, allowing for timely intervention and support.

Public health systems should consider policies that accommodate the recurring nature of PTSD symptoms over time. Screening conducted at six weeks, six months, and one year after the accident may be required to detect late-onset or persistent PTSD symptoms. Surveillance systems should also track people who experience minor injuries or report no physical injury, as psychological trauma can occur independently of physical damage [12,75,142].

Rehabilitation policies that prioritize psychological well-being alongside physical recovery are also needed [11]. Practitioners in general practice, emergency care, allied health, and insurance claim processes should receive training in trauma-informed care, with protocols adapted for early intervention in PTSD. Such workforce development is particularly important where mental health services are under-resourced despite high injury prevalence [12,13].

Post-accident care pathways should be diversified to include accessible and cost-effective interventions such as brief-CBT and emerging digital treatments like virtual reality exposure therapy, which have shown efficacy in reducing PTSD symptoms after RTA [74,125]. These interventions should be included in public health service delivery, especially where waitlists for long-term psychological care exceed demand.

Where PTSD symptoms lead to extended distress or functional impairment, links between mental health services and compensation schemes should be strengthened to ensure that psychological injuries are recognized and treated with parity to physical injuries. Access to psychological treatment and assessments may also need to be provided during and after compensation processes, particularly where fault or legal proceedings prolong stress responses [75,81].

Social support plays an essential role in recovery, yet it is inconsistently included in service planning. Public and mental health services should incorporate family-inclusive options and relationship counselling for those who report separation or relational distress post-RTA [64]. Where relevant, trauma-informed support for spouses and children should also be offered.

Public health education campaigns could reduce stigma and promote help-seeking behaviour. These campaigns should target groups vulnerable to underreporting or misrecognition of PTSD, such as men, individuals involved in minor RTAs, or those from low socioeconomic backgrounds. Messaging that validates “invisible” injuries can normalize psychological symptoms and increase access to early support.

Finally, mental health service planning should be informed by routinely collected data on post-RTA psychological outcomes. This includes data sharing between emergency departments, general practice, insurance providers, compensation bodies, and mental health services. Cross-sectoral data coordination can support timely referrals, track outcomes, and enable responsive policy development tailored to the mental health needs of RTA survivors. Without such coordination, mental health problems arising from RTAs risk being overlooked, under-treated, or misdiagnosed.

## 6. Limitations of the Extant Literature

Despite the strengths of this systematic review, several limitations must be acknowledged, and important directions for future research are outlined below. This review included studies published in multiple languages, enhancing inclusivity and allowing the capture of underrepresented populations in the PTSD literature. While this multilingual scope may reflect regional research priorities, the review also included studies from diverse global regions, including Europe, Asia, Africa, Australia, and North America. Therefore, although some regional healthcare and legal systems may differ, the findings reflect a broader international perspective compared to previous reviews that focused mainly on high-income, English-speaking countries. Still, the uneven geographic origin of publications may affect the generalizability of findings to populations in lower-income or non-English-speaking regions, highlighting the need for further research in diverse cultural contexts to better understand the psychological impacts of road traffic accidents globally.

Many studies in this literature review fail to report whether participants in the research were attending psychotherapies known to improve psychological health. Research is needed to understand the impact of social support, interventions, counselling, group sessions, or other kinds of support on the ongoing well-being of RTA survivors. An important finding of the literature review is that RTA survivors with minor injuries are not immune to PTSD, but unfortunately, little is known about the severity or longevity and the impact on their lives. The literature explores different interventions for the treatment of PTSD after RTA, but all had small numbers of participants, and results may not be generalizable to a larger population. In addition to using convenience sampling, cross-sectional designs, and self-report measures, further limitations should be acknowledged. Missing data and the associated reduction in sample sizes for some analyzes may have limited the statistical power to detect significant associations. Additionally, given the inclusion of studies published in multiple languages, there is potential for translation bias, which may have influenced the consistency and comparability of findings across different cultural contexts. Moreover, while many studies showed strong PTSD effects following RTAs, several studies reported non-significant or unexpected findings (e.g., no association between physical injury severity and PTSD severity). These inconsistencies highlight the need for cautious interpretation and suggest that factors beyond physical trauma, such as perceived threat, coping mechanisms, and pre-existing vulnerabilities, may play a greater role than initially assumed.

## 7. Conclusions

The reviewed literature considered people who experienced symptoms of post-traumatic stress disorder (PTSD) caused by a road traffic accident (RTA). The extant literature can be understood through a focus on the prevalence of PTSD, predictors of PTSD, management of thoughts, quality of life, post-traumatic growth, comorbidity of PTSD, treatments for PTSD, and the scales used to measure PTSD.

PTSD is prevalent in those surviving a road traffic accident, with nearly half of all RTA survivors experiencing PTSD six weeks after an RTA and over half of those initially diagnosed still experiencing PTSD up to three years after the RTA. People in minor traffic accidents are not exempt since 25% report avoiding using their car, motorcycle, or bicycle for up to 4 months after an RTA. Data has shown that people can recover from PTSD between 1 and 3 years after the RTA. However, the literature does not examine the cause of recovery, whether people received psychological evaluations and treatments (psychotherapy), and additional social and family support. While several treatments, such as CBT, VR, and Mem-Flex, have shown statistically significant effectiveness in reducing PTSD symptoms (e.g., *p* < 0.05), further large-scale trials are required. In addition, greater attention is needed to improve consistency in outcome reporting, longitudinal follow-up, and inclusion of minor injury cases across global contexts.

People severely injured in an RTA had significantly higher rates of separation from their partner compared to people less severely injured, with other sociodemographic characteristics significantly related to the presence of PTSD including female gender, below-average self-perceived economic status, medication use, psychiatric medication use, psychiatric illness history, road traffic accident injury affliction, injury severity, a self-perceived threat to life, post-road traffic accident pain, hospitalization duration, not at fault in road traffic accident and claiming compensation.

The severity of PTSD can be predicted by the severity of injuries immediately after the RTA. However, the severity of immediate injuries does not affect recovery from PTSD, and after one year no differences were reported in symptoms compared with people receiving only minor injuries. This literature review posits that traffic accident survivors with minor injuries are not immune to PTSD, but to date, little is known about the severity or longevity of their symptoms, and the impact of PTSD on health services and the lives of survivors.

Cognitive Behavioural Therapy (CBT) variants have been seen to reduce PTSD and have a positive impact on the mental health of the survivors of RTAs. Compared with other interventions, significantly more RTA survivors with PTSD experienced improvements after a 4-week Brief-CBT intervention than those in a self-help booklet program (SHP). Virtual Reality treatment has been seen to positively impact the psychological condition of people who were exposed to a traumatic event. However, writing about a stressful event may lead to more anxiety, and counselling as an intervention did not show effectiveness in preventing disorders after an RTA. There is consistency in the extant literature in the scales used to measure symptoms of PTSD. The Chinese version of the PTSD Checklist has been confirmed to have satisfactory reliability and validity. The Depression Anxiety Stress Scales (DASS-21) and the Impact of Event Scale-Revised (IES-R) have acceptable sensitivity and specificity for detecting Major Depressive Disorder and PTSD. However, the Glover Numbing Scale (GNS) is a poor fit for RTA survivors since some items showed only small standardized factor loadings. Unfortunately, existing measures are imperfect, as 25% of participants have been able to fake symptoms of psychological injury from a RTA in their psychological evaluation.

Future research should focus on improving the recording of symptoms of PTSD post-RTA, and the quality of the measures themselves, to elucidate the interplay between PTSD and these underexplored factors to provide a comprehensive understanding of post-RTA psychological experiences and inform targeted interventions.

## 8. Recommendations for Research

Future research on road traffic accidents (RTAs) and their psychological impacts should focus on several key areas to advance knowledge and inform effective interventions.

First, there is a critical need to explore the psychological impacts of RTAs in regions with high incidents of accidents. This exploration will provide a more comprehensive understanding of the global burden of post-accident trauma, especially in low-income countries (LICs) where research in this area is lacking.

Second, researchers should prioritize investigating psychological treatments for RTA survivors. These studies should systematically report treatment inclusion in methodologies to enhance transparency and comparability across studies. Additionally, a balanced representation of hospitalized and non-hospitalized participants should be included to capture the full spectrum of post-RTA psychological experiences and improve the generalizability of findings.

Third, standardization of PTSD assessment tools is essential to facilitate comparisons across studies and enhance the reliability and validity of findings. By adopting standardized assessment protocols, researchers can improve the accuracy and consistency of PTSD diagnoses, thereby advancing our understanding of post-RTA psychological outcomes.

Fourth, the presence of significant dissociative symptoms, such as those observed in Acute Stress Disorder (ASD) [40], along with the early prediction of PTSD using tools like the Trauma Screening Questionnaire (TSQ) [126] and Life Events Checklist (LEC) [114], has shown promise in other settings in predicting a subsequent diagnosis of PTSD. However, their applicability in the context of road traffic accidents has yet to be thoroughly explored.

Finally, while existing literature has extensively examined the relationship between PTSD and other psychological factors in RTA survivors, there is a need to explore additional psychosocial dimensions such as quality of life and social support.

## 9. Recommendations for Policy

The findings of this systematic review underscore the urgent need for public health and transport safety policies to address the psychological aftermath of road traffic accidents (RTAs). Based on the evidence of high and long-lasting prevalence of post-traumatic stress disorder (PTSD) among RTA survivors, including those with minor injuries, several policy-level actions are recommended:

### 9.1. Routine Psychological Screening Post-RTA

Health systems should implement standardized psychological screening protocols for all individuals involved in RTAs, regardless of injury severity. Screening should occur at multiple time-points—six weeks, six months, and one year post-accident—to detect both acute and delayed onset PTSD.

### 9.2. Integration of Brief CBT into Early Intervention Pathways

Given the demonstrated effectiveness of Brief Cognitive Behavioural Therapy (Brief-CBT), it should be integrated into early post-accident care pathways, particularly for individuals presenting with initial symptoms of trauma. This should be publicly funded and made accessible through primary care and telehealth services.

### 9.3. Public Awareness Campaigns Targeting Minor RTA Survivors

Awareness efforts should communicate that PTSD is not limited to major trauma survivors. Campaigns could reduce stigma, promote early help-seeking, and validate the experiences of individuals with “invisible injuries” following minor accidents.

### 9.4. Development and Regulation of PTSD Assessment Tools

Funding and policy support are needed to refine PTSD assessment tools that are resistant to response bias. Tools such as the PTSD Checklist and DASS-21 should be explicitly calibrated for RTA populations. Poorly fitting tools like the Glover Numbing Scale should be phased out.

### 9.5. Cross-Sector Data Sharing and Support Coordination

Efforts should be made to enhance data sharing between emergency services, insurance providers, health systems, and compensation bodies, enabling coordinated psychological care. This includes tracking injury severity, fault status, and claims processes, which correlate with PTSD outcomes.

### 9.6. Investment in Innovative Treatments, Including VR

Emerging evidence supports the potential of Virtual Reality Exposure Therapy (VRET) in PTSD treatment. Investment in trials, development, and eventual subsidization of VRET should be pursued to diversify treatment options.

### 9.7. Support for Families and Social Networks

Given the increased risk of relationship breakdown and the role of social support in recovery, post-RTA interventions should include optional family-inclusive support programs.

## Figures and Tables

**Table 1:** Study characteristics and LEGEND assessment [17].

Study	Year	Country/Region	LEGEND	Study Design	Primary Methodology	Notes
Adawi et al. [18]	2019	Italy	3B	Psychometric	Quantitative	Brief Symptom Inventory in nomophobic subjects
Angerpointner et al. [19]	2020	Germany	3B	Prospective	Quantitative	PTSD after minor trauma
Asmundson et al. [20]	1999	Canada	3B	Psychometric Study	Quantitative	Accident Fear Questionnaire validation
Asuquo et al. [21]	2017	Nigeria	4B	Case-control	Quantitative	PTSD vs. controls post-RTA
Aykaç et al. [22]	2024	Turkey	4A	Cross-sectional	Quantitative	Post-traumatic growth after RTA
Bae et al. [23]	2014	Korea	3B	Cross-sectional	Quantitative	Memory function and PTSD
Bae et al. [24]	2015	Korea	4B	Cross-sectional	Quantitative	Forgiveness as PTSD mediator
Baptie et al. [25]	2021	UK	2B	RCT	Mixed Methods	VR intervention for trauma recovery
Barth et al. [26]	2005	Germany	3B	Longitudinal	Quantitative	PTSD and psychosocial impairments
Beck et al. [27]	2006	USA	3B	Experimental	Quantitative	Thought suppression post-RTA
Beck et al. [28]	2007	USA	4B	Case Series	Mixed Methods	Virtual Reality Exposure Therapy
Bedaso et al. [29]	2020	Ethiopia	3B	Prospective	Quantitative	PTSD prevalence among survivors
Benjamin et al. [30]	2010	USA	3B	Experimental	Quantitative	Subjective distress in anxious youth
Berntsen & Rubin [31]	2006	USA	3A	Psychometric	Quantitative	Centrality of event scale for trauma
Blanchard et al. [32]	1996	USA	3B	Prospective	Quantitative	PTSD remission after 1 year
Blanchard et al. [33]	2003	USA	3B	Experimental	Quantitative	Prediction of response to therapy
Blanchard & Buckley [34]	1998	USA	3B	Prospective	Quantitative	Psychophysiological changes after RTA
Blanchard & Hickling [35]	2004	USA	3B	Book	Qualitative	Assessment and treatment post-MVA
Boccia et al. [11]	2015	Italy	1B	Meta-analysis	Quantitative	EMDR treatment efficacy for RTA survivors
Boelen et al. [36]	2024	Netherlands	3B	Survey	Mixed Methods	Preferences for PTSD treatment modes
Brand et al. [37]	2014	Germany	3A	Cross-sectional	Quantitative	PTSD incidence after RTAs
Briere et al. [38]	1995	USA	4A	Psychometric	Quantitative	Trauma Symptom Inventory validation
Brom et al. [39]	1993	Netherlands	3B	Cross-sectional	Mixed Methods	Counselling outcomes post-RTA
Bryant et al. [40]	2000	Australia	4A	Prospective	Quantitative	Acute stress predicting PTSD
Bryant et al. [41]	2021	Australia	2A	RCT	Mixed Methods	Brief vs. long CBT exposure therapies
Buckby et al. [42]	2007	Australia	3B	Psychometric	Quantitative	MASQ validation in youth
Carroll et al. [43]	2010	USA	3B	Review	Quantitative	AIS revision for brain injury research
Choi et al. [44]	2017	South Korea	3B	Psychometric	Quantitative	Coping Inventory validation
Chossegros et al. [45]	2011	France	3A	Cohort	Quantitative	PTSD at 6 months post-RTA
Clapp & Beck [46]	2009	USA	3B	Psychometric	Quantitative	Glover Numbing Scale examination
Coles et al. [47]	2014	USA	4A	Psychometric	Quantitative	Evaluation of Sheehan Disability Scale
Coronas et al. [48]	2008	Spain	4B	Cross-sectional	Quantitative	PTSD onset in RTA survivors
Daddah et al. [12]	2022	Benin	3B	Cross-sectional	Quantitative	PTSD risk factors post-RTA
Daneshvar et al. [49]	2021	Iran	3B	Experimental	Quantitative	Positive emotion intervention on memory
Davidson et al. [50]	2002	USA	4A	Psychometric	Quantitative	Norms for Davidson Trauma Scale
Ehring et al. [51]	2008	UK	3B	Experimental	Quantitative	Memory updating in PTSD
Ehring & Ehlers [52]	2014	UK	4A	Experimental	Quantitative	Rumination and PTSD symptoms
Fecteau & Nicki [53]	1999	Canada	2B	RCT	Quantitative	CBT for PTSD post-MVA
Fedroff et al. [54]	2000	Canada	4A	Experimental	Quantitative	CBT impact on anxiety sensitivity
Fekadu et al. [55]	2019	Ethiopia	3B	Cross-sectional	Quantitative	PTSD impacts on work and life
Feki et al. [56]	2024	Tunisia	4B	Cross-sectional	Quantitative	Gender differences in PTSD risk
Figlerski et al. [57]	1989	USA	3B	Survey	Quantitative	Anxiety after minor RTAs
Fort et al. [58]	2018	France	4A	Prospective	Quantitative	Return to work predictors post-RTA
Ghanizadeh & Tavassoli [59]	2007	Iran	3B	Community Study	Quantitative	Adolescents’ PTSD symptoms
Gidron et al. [60]	2001	Israel	3B	RCT (pilot)	Mixed Methods	Memory intervention (Mem-Flex)
Gittins et al. [61]	2015	UK	3B	RCT	Quantitative	Writing about trauma vs. non-recall task
Guest et al. [26]	2018	Australia	4B	Psychometric	Quantitative	DASS-21 and IES-R post-RTA
Hekimoglu et al. [62]	2022	Turkey	3B	Longitudinal	Quantitative	PTSD prevalence at 7 years post-RTA
Holeva et al. [63]	2001	UK	3A	Cross-sectional	Quantitative	Acute stress and social support
Hours et al. [64]	2013	France	3A	Longitudinal	Quantitative	Severe injury and relational impact
Hovens & van de Weerd [65]	1998	Netherlands	3B	Prospective	Quantitative	Hyperarousal predicting PTSD
Iyadurai [66]	2015	UK	2B	RCT	Quantitative	Cognitive task to reduce intrusive memories
Jeavons [67]	2000	UK	3B	Prospective	Quantitative	PTSD and subsyndromal PTSD at 1 year
Jones et al. [68]	2005	UK	3A	Prospective	Quantitative	TBI and PTSD prediction
Jones et al. [69]	2007	UK	2B	Qualitative	Mixed Methods	Trauma memory organization post-RTA
Keshet et al. [70]	2019	Israel	4B	Cross-sectional	Quantitative	Self-perceptions post-RTA trauma
Kessler et al. [71]	2021	USA	4A	Prospective	Quantitative	Trauma predictors of PTSD post-MVA
Khati et al. [72]	2013	France	2B	Cohort	Quantitative	Quality of life post-RTA
Khodadadi-Hassankiadeh et al. [73]	2017	Iran	3A	Cross-sectional	Quantitative	Depression, amnesia, PTSD
Kim et al. [74]	2022	Korea	3A	Cohort	Mixed Methods	VR Exposure Therapy vs. control group
Koren et al. [7]	2001	Israel	4A	Longitudinal	Quantitative	Chronic PTSD over 3 years
Kovacevic et al. [75]	2020	Croatia	3A	Cross-sectional	Quantitative	Predictors of PTSD and mental health
Kramer et al. [76]	2005	Switzerland	4B	Cross-sectional	Quantitative	Dyadic coping post-trauma
Krzemieniecki & Gabriel [77]	2021	USA	3A	Survey	Quantitative	Stigma and trauma type comparison
Leroy et al. [6]	2022	France	2B	Cohort	Quantitative	PTSD prevalence after 1 year
Li et al. [78]	2021	China	4A	Prospective	Quantitative	Acute Stress Disorder predicting PTSD
Lin et al. [9]	2018	China	3B	Meta-analysis	Quantitative	PTSD prevalence meta-analysis
Lowinger & Solomon [79]	2004	Israel	3B	Cross-sectional	Quantitative	Self-blame in reckless drivers
Mairean [80]	2019	Romania	3A	Survey	Quantitative	Driving cognitions and rumination
Matthews [81]	2005	Australia	3B	Cross-sectional	Quantitative	Work limitations with PTSD post-RTA
Mayou et al. [82]	2000	UK	3B	Longitudinal	Quantitative	Unconsciousness and PTSD symptoms
Medhaffar et al. [83]	2016	Tunisia	4A	Cross-sectional	Quantitative	Injury type and PTSD development
Miller et al. [84]	1995	USA	3B	Psychometric	Quantitative	MMPI-2 PTSD scale assessment
Mirabolfathi et al. [49]	2019	Iran	4A	Experimental	Quantitative	Affective distractors and memory
Murray et al. [85]	2002	UK	3A	Prospective	Quantitative	Injury severity predicting PTSD
Nightingale & Williams [86]	2000	UK	4A	Cross-sectional	Quantitative	PTSD and emotional expression
Nilsen et al. [87]	2016	Norway	3B	fMRI Study	Quantitative	Brain activation in PTSD post-RTA
Olaya et al. [88]	2015	Spain	3B	Survey	Quantitative	Sociodemographic predictors of PTSD
Piltan et al. [89]	2021	Iran	2A	Experimental	Quantitative	Memory flexibility and PTSD
Ponniah & Hollon [10]	2009	USA	2A	Review (of RCTs)	Quantitative	Treatment efficacy for PTSD
Rabe et al. [90]	2006	Germany	2A	Physiological Study	Quantitative	Heart rate reactivity and PTSD
Ramirez et al. [91]	2001	USA	4A	Survey	Quantitative	Numbing, apathy, and depression
Ratnani [92]	2022	India	3A	Cross-sectional	Quantitative	PTSD prevalence within 1 year
Roitman et al. [93]	2013	Israel	3B	Cross-sectional	Quantitative	Unconsciousness and PTSD risk
Roussis [94]	2007	UK	3B	Thesis	Quantitative	Metacognitive factors in PTSD
Sadeghi-Bazargani et al. [95]	2022	Iran	4A	Cohort	Quantitative	PTSD prevalence at 1 month post-RTA
Sescosse et al. [96]	2018	USA	2B	RCT	Quantitative	Post-traumatic growth and recovery
Smith et al. [5]	1997	UK	3A	Prospective	Quantitative	PTSD prevalence at 6 weeks post-RTA
Sun et al. [97]	2015	China	4B	Neuroimaging Study	Quantitative	Corpus callosum changes in PTSD
Veazey et al. [98]	2004	USA	5A	Psychophysiological	Quantitative	Heart rate in PTSD survivors
Walshe et al. [99]	2015	China	3B	Cohort	Quantitative	Art therapy for PTSD post-RTA
Wan et al. [100]	2015	China	3B	RCT	Mixed Methods	Art therapy for PTSD post-RTA
Wu et al. [101]	2014	China	2A	RCT	Mixed Methods	CBT vs. self-help booklet intervention
Yasan et al. [102]	2019	Turkey	3A	Longitudinal	Quantitative	PTSD trajectory over a year
Yimer et al. [13]	2023	Ethiopia	3B	Cross-sectional	Quantitative	PTSD comorbidity rates
Zoellner et al. [103]	2008	Switzerland	4A	Cross-sectional	Quantitative	Post-traumatic growth and PTSD

**Table 2:** Study characteristics—country of first author of publication and year of publication.

Country of Publication (*n* = 96)	
Country of publication	Number of studies
USA	22
UK	13
Iran	6
China	6
France	5
Australia	5
Israel	5
Germany	5
Netherlands	3
Korea	3
Ethiopia	3
Turkey	3
Canada	3
Switzerland	2
Tunisia	2
Italy	2
Spain	2
Benin	1
Croatia	1
South Korea	1
Nigeria	1
Romania	1
Norway	1
India	1
**Year of publication**	**Number of studies (%) (*n* = 96)**
−1999	10 (10.4%)
2000–2010	34 (35.4%)
2011–2024	54 (56.2%)

**Table 3:** Sample characteristics—inclusion of psychological treatments and hospitalization post-RTA.

Sample Characteristics	
**Number of participants included in all studies**	50,275
**Information about psychological treatments**	**Number of studies (%) (*n* = 96)**
No information about participants having any kind of psychological treatment after the accident	77 (80.2%)
Studies that report that participants have some psychological treatment after the accident	21 (21.9%)
**Patient type**	**Number of studies (%) (*n* = 96)**
Hospitalized after a Road Traffic Accident	31 (32.3%)
Not hospitalized after the Road Traffic Accident	67 (69.8%)

**Table 4:** Study characteristics—PTSD scales used by studies from the systematic review.

Used Scales for PTSD (Original Author)	Number (%) of Studies (*n* = 96)	Studies Using the Scale
PTSD Checklist-(PCL-5) [107]	26 studies (27.1%)	[4,5,6,12,13,29,30,34,45,56,64,75,77,78,80,81,84,89,90,93,95,96,107,108,109,110]
Clinician-Administered PTSD Scale (CAPS) [111]	24 studies (25%)	[4,5,6,12,18,21,44,49,58,63,71,74,78,82,105,106,107,112,113,114,115,116,117,118]
PTSD Symptom Scale—Interview, PSS-I-5 scale [119]	15 studies (15.6%)	[27,36,56,67,68,69,71,73,76,81,82,93,96,108,120]
PTSD Symptom Scale Self-Report Version (PSS-SR) [121]	4 studies (4.2%)	[7,54,65,86]
Post-traumatic Diagnostic Scale [5]	3 studies (3.1%)	[70,85,122]
Mississippi PTSD Scale for Civilians [112]	2 studies (2.1%)	[50,60]
Davidson Trauma Scale (DTS) [123]	1 study (1%)	[120]

**Table 5:** Study characteristics—other scales used in the studies in the systematic review.

Used Scales (Original Author)	Number (%) of Studies (*n* = 96)	Studies Using the Scale
The Impact of Event Scale (Revised IES-R) [124]	17 studies (17.7%)	[7,23,31,34,36,55,71,75,92,97,103,108,111,119,124,125,126]
Beck depression and anxiety inventories [124]	9 studies (9.4%)	[7,19,23,27,39,49,53,57,61,67,74,94,97,100,101,124,127]
Motor Vehicle Accident Interview (MVA) Interview [36]	4 studies (4.2%)	[33,46,54,63]
Post Traumatic Growth Inventory [128]	4 studies (4.2%)	[19,22,34,96]
The State-Trait Anxiety Inventory (STAI) [129]	3 studies (3.1%)	[25,33,98]
The Peritraumatic Dissociative Experiences Questionnaire [130]	2 studies (2.1%)	[102,124]
Anxiety Sensitivity Index [131]	1 study (1%)	[61]
Mayer-Salovery-Caruso Emotional Intelligence Test (MSCEIT) [115]	1 study (1%)	[46]
The Minnesota Multiphasic Personality Inventory-2 (MMPI-2) [132]	1 study (1%)	[64]
The Multidimensional Scale of Perceived Social Support [133]	1 study (1%)	[23]
Composite International Diagnostic Interview (CIDI) [116]	1 study (1%)	[41]
The Depression Anxiety Stress Scales—21 (DASS-21) [117]	1 study (1%)	[61]
Post event information (PEI) [134]	1 study (1%)	[61]
Coping Inventory for Stressful Situations [45]	1 study (1%)	[68]
World Assumption Scale [135]	1 study (1%)	[67]
The Centrality of Event Scale [32]	1 study (1%)	[70]
The Post-traumatic Cognitions Inventory (PTCI) [136]	1 study (1%)	[70]
The Driving Cognitions Questionnaire [114]	1 study (1%)	[80]
The visual analog rating scale of health-related quality of life [137]	1 study (1%)	[107]
Mood and Anxiety Symptom Questionnaire (MASQ) [43]	1 study (1%)	[107]
Trait Coping Style Scale [138]	1 study (1%)	[119]
The Abbreviated Injury Scale (AIS) [44]	1 study (1%)	[119]
Glover Numbing Scale [92]	1 study (1%)	[46]
Injury Severity Score [139]	1 study (1%)	[103]
The Longitudinal Interval Follow-up Evaluation (LIFE) [140]	1 study (1%)	[33]
Brief Symptom Inventory [19]	1 study (1%)	[33]
The Social Support Questionnaire [118]	1 study (1%)	[63]
The Thought Control Questionnaire [141]	1 study (1%)	[63]
The Accident Fear Questionnaire [21]	1 study (1%)	[53]
Trauma Symptom Inventory [39]	1 study (1%)	[39]
The quality of life questionnaire [142]	1 study (1%)	[95]
Oslo social support scale (OSSS-3) [125]	1 study (1%)	[13]
Sheehan Disability Scale [48]	1 study (1%)	[13]
Scale of Psychological Well-being [113]	1 study (1%)	[96]

## Data Availability

The data will be made available on reasonable request to the corresponding author.
